# Targeted nanopore sequencing using clinical specimens for the rapid diagnosis of extrapulmonary tuberculosis

**DOI:** 10.1186/s12879-024-09618-0

**Published:** 2024-07-19

**Authors:** Guocan Yu, Likui Fang, Yanqin Shen, Fangming Zhong, Xudong Xu

**Affiliations:** Zhejiang Tuberculosis Diagnosis and Treatment Center, Hangzhou Red Cross Hospital, Hangzhou, Zhejiang China

**Keywords:** Extrapulmonary tuberculosis, Targeted nanopore sequencing, Lymph node tissue, Cerebrospinal fluid, Pleural effusion, Accuracy, Sensitivity

## Abstract

**Background:**

The clinical presentation of extrapulmonary tuberculosis (EPTB) is atypical and it is easily confused with other diseases such as common infections, making prompt diagnosis a great challenge. This study aimed to evaluate the accuracy of targeted nanopore sequencing (TNS) in the diagnosis of EPTB. The diagnostic accuracy of TNS using different types of extrapulmonary specimens was also evaluated.

**Methods:**

We reviewed the clinical data of patients with suspected EPTB for whom TNS was conducted and who were hospitalized at our center. The true positive, false positive, false negative, and true negative values were determined. Indices of diagnostic accuracy were computed, including sensitivity, specificity, positive predictive value (PPV), negative predictive value (NPV), and area under the curve (AUC) for TNS and acid-fast bacilli (AFB) culture, and compared with those from clinical diagnosis.

**Results:**

149 patients were included in the analysis. The overall sensitivity, specificity, PPV, NPV, and AUC of TNS for the diagnosis of EPTB were 86.4%, 87.5%, 97.3%, 55.3%, and 0.87, respectively. For diagnosis by AFB culture, these values were 25.6%, 100.0%, 100.0%, 20.5%, and 0.63, respectively. The most common specimens used were lymph node tissue, cerebrospinal fluid, pleural effusion, and pleural tissue. The diagnostic accuracy of TNS using all types of extrapulmonary specimens was good.

**Conclusions:**

TNS demonstrates good diagnostic accuracy in the rapid diagnosis of EPTB and this was true across different types of extrapulmonary specimens.

## Background

Human infection with *Mycobacterium tuberculosis* (MTB) and the resultant tuberculosis (TB) is a major global public health issue, especially in developing countries [1]. In 2022, about 10.6 million people worldwide were diagnosed with TB and about 1.3 million people died from TB. China is the third highest TB burden country, with about 748,000 new cases of TB and 30,000 deaths due to TB in 2022 [1]. TB may be broadly classified as pulmonary TB (PTB) or extrapulmonary TB (EPTB) [1]. EPTB accounts for approximately 20% of cases and infection can occur at numerous sites, with the most common forms being lymph node TB, bone and joint TB, TB pleurisy, and TB meningitis [2, 3]. The increased prevalence of immunodeficiency diseases has led to a corresponding increase in the incidence of EPTB [4]. The clinical presentation of EPTB is atypical and it is easily confused with other diseases such as common infections, making prompt diagnosis a great challenge [5]. Meanwhile, the acquisition of EPTB specimens usually requires invasive procedures that may involve some risks, which increases the difficulty of EPTB diagnosis [6].

Classical acid-fast bacilli (AFB) smear and AFB culture are the most common methods of EPTB diagnosis [7]. However, the majority of EPTB specimens are paucibacillary, making the positivity rate of AFB smear and AFB culture relatively low, with poor sensitivity to EPTB [8]. Furthermore, it takes time to obtain the results of AFB culture, preventing early diagnosis of EPTB [3]. Hence, research into more effective diagnostic methods is ongoing.

Molecular detection techniques allow rapid detection of pathogens and are an important advance in TB diagnosis, including EPTB [9, 10]. Nanopore sequencing is a new method of single-molecule, real-time sequencing, which detects the current changes of single-molecule DNA (RNA) through biological nanopores to infer the base composition, with long read length, fast speed, real-time monitoring, and portable features [11, 12]. It has demonstrated excellent diagnostic accuracy in the early diagnosis of PTB, significantly better than the current gold standard (AFB culture) [13]. However, its accuracy in the diagnosis of EPTB remains unevaluated.

Nanopore sequencing can be performed without amplification of target pathogens, which may affect its diagnostic accuracy when the level of target pathogens is extremely low, in order to solve this challenge, targeted nanopore sequencing (TNS) came into being. TNS uses polymerase chain reaction (PCR) to amplify the target pathogens DNA or RNA prior to nanopore testing, which increases the level of the target pathogens, which in turn improves the sensitivity of nanopore sequencing [14]. This is of particular value with diseases in which specimens are prone to low pathogen content, such as EPTB. This study aimed to evaluate the accuracy of TNS in the diagnosis of EPTB and to identify any variations in its diagnostic accuracy between different types of extrapulmonary specimens.

## Methods

### Study design

This retrospective study was conducted at a provincial regional TB diagnosis and treatment center. We reviewed the clinical data of patients tested with TNS for suspected EPTB at our center between September 2021 to October 2022. The data were obtained from our electronic medical records. EPTB is suspected in patients with TB-related symptoms such as low-grade fever, night sweats, abnormal signs at the site of onset (e.g., enlarged lymph nodes), pleural and abdominal fluid, positive TB immunoassays (purified protein derivative [PPD] test and/or gamma interferon release assay), imaging test results from ultrasound, computed tomography, or magnetic resonance imaging suggestive of possible TB (e.g., ring enhancement of lymph nodes or bone destruction), biochemical effusion test results suggestive of possible TB (e.g., elevation of adenosine deaminase [ADA] levels), granulomatous inflammation with necrosis found in pathology tests, and/or symptom reduction resulting from anti-TB therapy.

Patients with suspected EPTB whose extrapulmonary specimens were tested using both TNS and AFB culture and were followed up for at least 9 months were included in the study. Patients for whom extrapulmonary specimens were not used for relevant testing, who were not tested using both TNS and AFB culture, and who were lost to follow-up were excluded. The types of extrapulmonary specimens are varied and commonly include tissue, pus, fluids, and urine. Any of these types of specimens obtained from any extrapulmonary organ can be included. Some extrapulmonary specimens were obtained using invasive procedures (such as thoracentesis, lumbar puncture, lymph node puncture). Written informed consent was obtained from the patient or his/her guardian for these invasive procedures. The study protocol was approved by the Ethics Committee of Hangzhou Red Cross Hospital (2023-YS-133). The requirement for informed consent to study inclusion and the need for consent to participate were waived by the Ethics Committee of Hangzhou Red Cross Hospital because of the lack of study intervention in patient diagnosis and treatment and the retrospective nature of the study. This study is in accordance with the Declaration of Helsinki.

Based on their eventual clinical diagnosis, we divided the patients in our sample into three groups for statistical analysis.

Group A (confirmed EPTB) comprised those in whom EPTB was confirmed with positive AFB culture from extrapulmonary specimens. Group B (probable EPTB) comprised those diagnosed with probable EPTB in whom clinical manifestations were observed, with positive results on a PPD and/or gamma interferon release assay, imaging results suggestive of TB, and/or elevated ADA levels and lymphocytic predominance in serous effusions, who showed a positive response to anti-TB treatment. Group C (non-EPTB) comprised those without EPTB whose AFB culture were negative for MTB, another diagnosis was established, or who improved without anti-TB treatment. All patients in groups A and B were clinically diagnosed with EPTB.

### Diagnostic specimen collection and handling

Two senior supervising physician performed invasive procedures (such as thoracentesis, abdominal puncture, lumbar puncture, lymph node puncture, and surgery) to collect relevant specimens. The fresh specimens were stored in a 4 °C refrigerator and tested within 2 h. Tissue specimens were pretreated by grinding, fluids specimens were pretreated by centrifugation, and pus specimens could be used directly; pretreated specimens were divided equally for culture (including AFB, bacteria, and fungi) and for use in the TNS. Puncture and tissue samples were also used for histopathological examination. Meanwhile, we collected basic information such as patient’s name, gender, height, weight, medication history, and other relevant details corresponding to the specimen.

### AFB culture technique

For AFB culture technique, a freshly collected sample was utilized. Effusion specimens can be used directly, while tissue or puncture specimens require grinding before use. Digestion and decontamination of clinical specimens using 4% N-acetyl-l-cysteine–NaOH to remove possible contaminants. Added 1–2 times the volume of N-acetyl-l-cysteine–NaOH to the specimen and shake using a vortex shaker to homogenize the specimen. 0.1 ml of the treated specimen was taken and aseptically inoculated on the medium. Two different growth media: Lowenstein–Jensen solid medium and BACTEC MGIT 960 liquid medium (BD Diagnostic Systems in Sparks, MD) were used for AFB culture. The inoculated media were placed in a 37 °C thermostat for incubation and observed weekly for colony growth [15]. If no colony growth was observed for 6 weeks, the culture was reported as negative for AFB.

### Nanopore sequencing

The procedure for the pretreatment of TNS specimens is the same as that for AFB culture specimens. The main TNS procedure consists of the following steps. NaOH solution-treated specimens are centrifuged to remove the supernatant and obtain the precipitate. The precipitate is washed and resuspended in phosphate-buffered saline. The solution obtained is then ground with grinding beads and further lysed through the addition of lysozyme solution. A QIAamp DNA microbiome kit (Cat. No. 51,707, Qiagen, Hilden, Germany) is then used to extract DNA from the lysis product.

The extracted DNA is amplified via polymerase chain reaction (PCR) with specific targeting of the ropB gene primers (Rpo5′ [5′-TCAAGGAGAAGCGCTACGA-3′] and Rpo3′ [5′-GGATGTTGATCAGGGTCTGC-3′]) for *Mycobacterium* spp. The touchdown method is used for the PCR. First, denaturation is performed at 98 °C for 3 min. Next, six cycles of amplification are implemented. Each cycle consists of denaturation at 95 °C for 15 s. This is followed by annealing at 66 °C for 60 s and elongation at 72 °C for 30 s. During elongation, the temperature is gradually decreased by 1 °C per cycle. After the initial six cycles, the annealing temperature is further lowered to 61 °C for 29 cycles. A final extension step is performed at 72 °C for 5 min to complete the PCR process.

The PCR amplification products are purified and labeled using the barcode method. The labeled products are subjected to nanopore sequencing using the GridION platform. After obtaining the sequences, a series of quality checks are performed to ensure the accuracy and reliability of the data. The sequences are filtered to eliminate any disreputable or irrelevant fragments. Then, using MiniMap 2 software, the filtered sequences are aligned with the reference sequences for MTB and *Mycobacterium* to identify and classify the microbial species present in the sample. To eliminate any possibility of contamination by the host, the DNA reads originating from the host (in this case, human) are specifically targeted and removed. This is achieved by aligning the sequences with the human reference genome (GRCh38) which serves as a benchmark for identifying host DNA [13]. Following this comprehensive process allows conclusive results to be obtained within just 48 h.

### Data processing and statistical analysis

Data were described as means, quartiles, and standard deviations and analyzed using SPSS v. 24.0 (IBM Corp., Armonk, NY, USA) software. The true positive, false positive, false negative, and true negative values were also determined using SPSS. To evaluate the diagnostic performance of the compared methods, several indices of diagnostic accuracy were computed; these were sensitivity, specificity, positive predictive value (PPV), negative predictive value (NPV), and area under the curve (AUC). These calculations were performed using MedCalc v.15.2.2 statistical software. For each of these accuracy indices, the 95% confidence intervals were determined.

To ensure the validity of the data, Levene’s test was used to assess the equality of variances between groups, and the Shapiro–Wilk test was used to examine the normality of the data distribution. Between-group differences of normally distributed data with equal variances were analyzed using t-tests. For non-normally distributed data, we used the Wilcoxon rank sum tests. Paired data were analyzed using McNemar’s test. Ratios between two groups were compared using either chi-square test or Fisher’s exact test. Differences between two AUC values were compared using z-tests. These analyses were conducted using SPSS. For all tests, a *p*-value of < 0.05 was considered statistically significant.

## Results

**Fig. 1:**
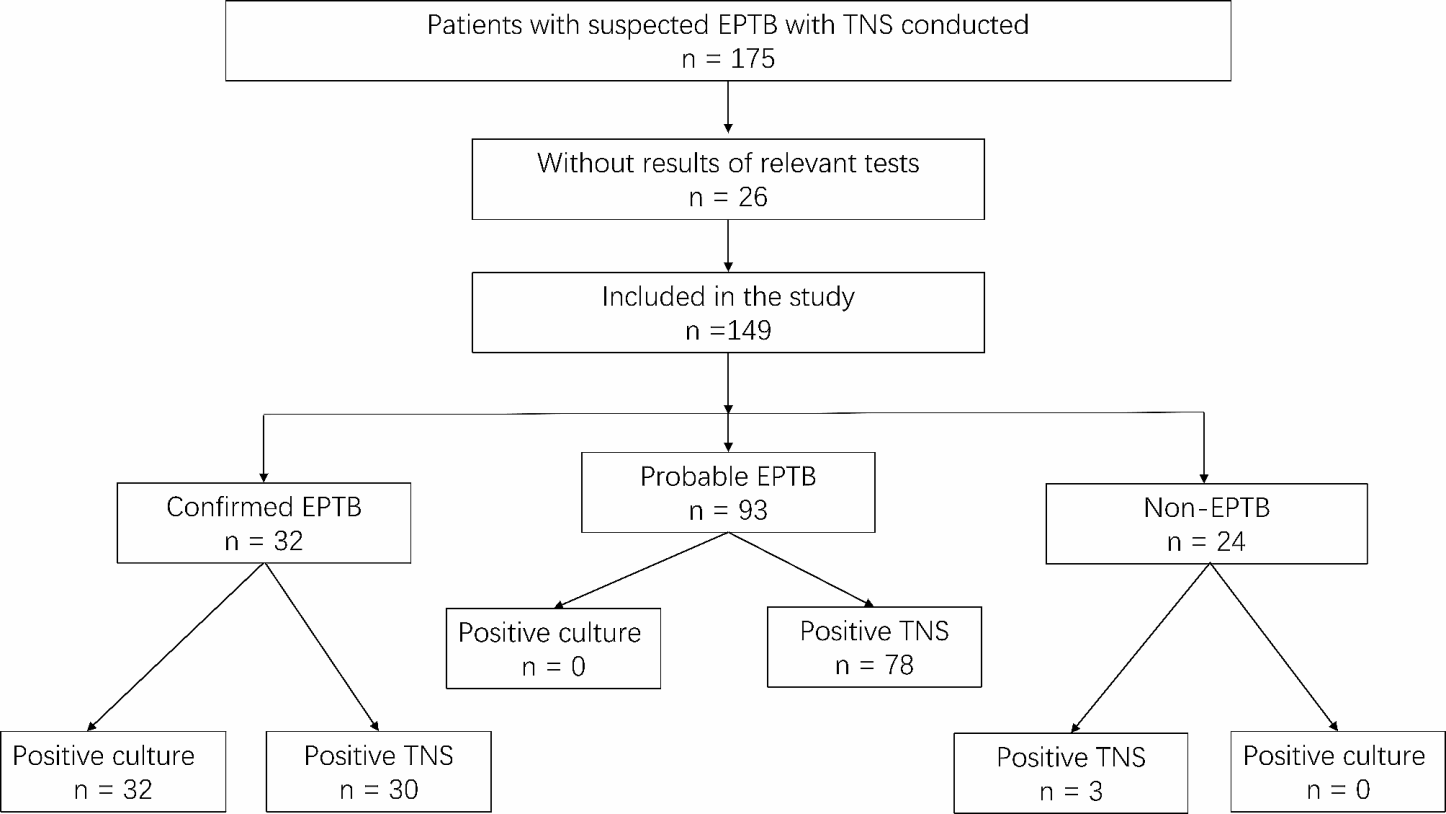
Screening process and diagnostic classifications for inclusion of patients

During the study period, 175 patients underwent TNS testing. Among these, the results of the AFB culture of 26 patients were not available. After the exclusion of these data, 149 patients remained and were included in our analysis. The patient screening process is displayed in Fig. 1. None of the patients in our sample were infected with HIV. Of the 149 patients, 83 (55.7%) were male, and the age range was 2–87 years, with an average age of 42.4 years. One extrapulmonary specimen was obtained from each patient. All patients were not on anti-tuberculosis treatment before the relevant tests were performed. In the non-EPTB group, we observed 1 case of cryptococcus, 1 case of escherichia coli, 2 cases of aspergillus, and 3 cases of non-tuberculous mycobacterial infections. Baseline characteristics of patients is presented in Table 1. The distribution of specimen types is shown in Table 2. The most common samples were lymph node tissue, cerebrospinal fluid, pleural effusion, and pleural tissue.

**Table 1:** Baseline characteristics of patients

Characteristics	All EPTB (*n* = 149)	Confirmed EPTB (*n* = 32)	Probable EPTB (*n* = 93)	Non-EPTB (*n* = 24)
Age (year, median, IQR)	40.0 (26.5–59.0)	31.0 (24.5–59)	33.0 (26.0-52.5)	59.0 (51.0-65.5)
Male (n, %)	83 (55.7)	22 (68.7)	49 (52.7)	12 (50.0)
Female (n, %)	66 (44.3)	10 (31.3)	44 (47.3)	12 (50.0)
HIV positive (n, %)	0 (0.0)	0 (0.0)	0 (0.0)	0 (0.0)
Weight (Kg, median, IQR)	55.0 (49.0-64.5)	54.5 (45.8–64.0)	55.0 (50.0-63.5)	57.0 (52.6–65.0)
Height (cm, median, IQR)	165 (160–171)	170 (161–175)	164 (160–171)	162 (159–170)
BMI (Kg/m^2^, median, IQR)	20.6 (18.5–23.0)	19.8 (17.6–21.1)	20.6 (18.6–22.8)	21.5 (20.0-24.4)
Laboratory examinations				
WBC (*10^9^/L, median, IQR)	4.8 (4.1–6.1)	5.2 (4.4–6.2)	4.9 (4.1–6.5)	4.1 (3.5–4.5)
RBC (*10^12^/L, median, IQR)	4.6 (3.9–5.2)	4.6 (3.9–5.5)	4.6 (3.9–5.2)	4.6 (3.7–4.9)
PLT (*10^12^/L, median, IQR)	222 (182–273)	226 (189–286)	223 (181–270)	189 (158–232)
ESR (mm/h, median, IQR)	22.5 (14.8–47.3)	27.0 (16.5–44.0)	19.0 (12.0–49.0)	18.0 (11.0–46.0)
CRP (mg/L, median, IQR)	7.8 (2.4–22.1)	16.8 (3.8–29.0)	8.0 (3.4–22.5)	2.1 (0.9–6.8)

EPTB: extrapulmonary tuberculosis; IQR: interquartile range; HIV: human immunodeficiency virus; BMI: body mass index; WBC: white blood cell; RBC: red blood cell; PLT: platelet; ESR: erythrocyte sedimentation rate; CRP: C-reactive protein

**Table 2:** The distribution of extrapulmonary specimen types

Sample type		All EPTB (*n* = 149)	Confirmed EPTB (*n* = 32)	Probable EPTB (*n* = 93)	Non-EPTB (*n* = 24)
Tissue	Abdominal tissue	1 (0.7)	0 (0.0)	1 (1.1)	0 (0.0)
	Bone tissue	2 (1.4)	0 (0.0)	2 (2.2)	0 (0.0)
	Lymph node tissue	36 (24.2)	10 (31.3)	19 (20.4)	7 (29.2)
	Pleural tissue	21 (14.1)	1 (3.1)	19 (20.4)	1 (4.2)
	Breast tissue	1 (0.7)	0 (0.0)	0 (0.0)	1 (4.2)
	Esophageal tissue	1 (0.7)	0 (0.0)	1 (1.1)	0 (0.0)
	Chest wall tissue	7 (4.7)	1 (3.1)	5 (5.4)	1 (4.2)
Pus	Liver pus	2 (1.4)	1 (3.1)	1 (1.1)	0 (0.0)
	Lymph node pus	4 (2.8)	0 (0.0)	4 (4.3)	0 (0.0)
	Pyothorax pus	4 (2.8)	0 (0.0)	3 (3.2)	1 (4.2)
	Skin pus	2 (1.4)	2 (6.2)	0 (0.0)	0 (0.0)
	Lumbar pus	1 (0.7)	1 (3.1)	0 (0.0)	0 (0.0)
Fluid	Ascitic fluid	4 (2.8)	1 (3.1)	2 (2.2)	1 (4.2)
	Joint effusion	1 (0.7)	0 (0.0)	0 (0.0)	1 (4.2)
	Cerebrospinal fluid	28 (18.8)	9 (28.1)	15 (16.1)	4 (16.7)
	Pericardial fluid	6 (4.0)	0 (0.0)	4 (4.3)	2 (8.3)
	Pleural fluid	22 (14.8)	5 (15.6)	15 (16.1)	2 (8.3)
Urine	Urine	6 (4.0)	1 (3.1)	2 (2.2)	3 (12.5)

EPTB, extrapulmonary tuberculosis

**Fig. 2:**
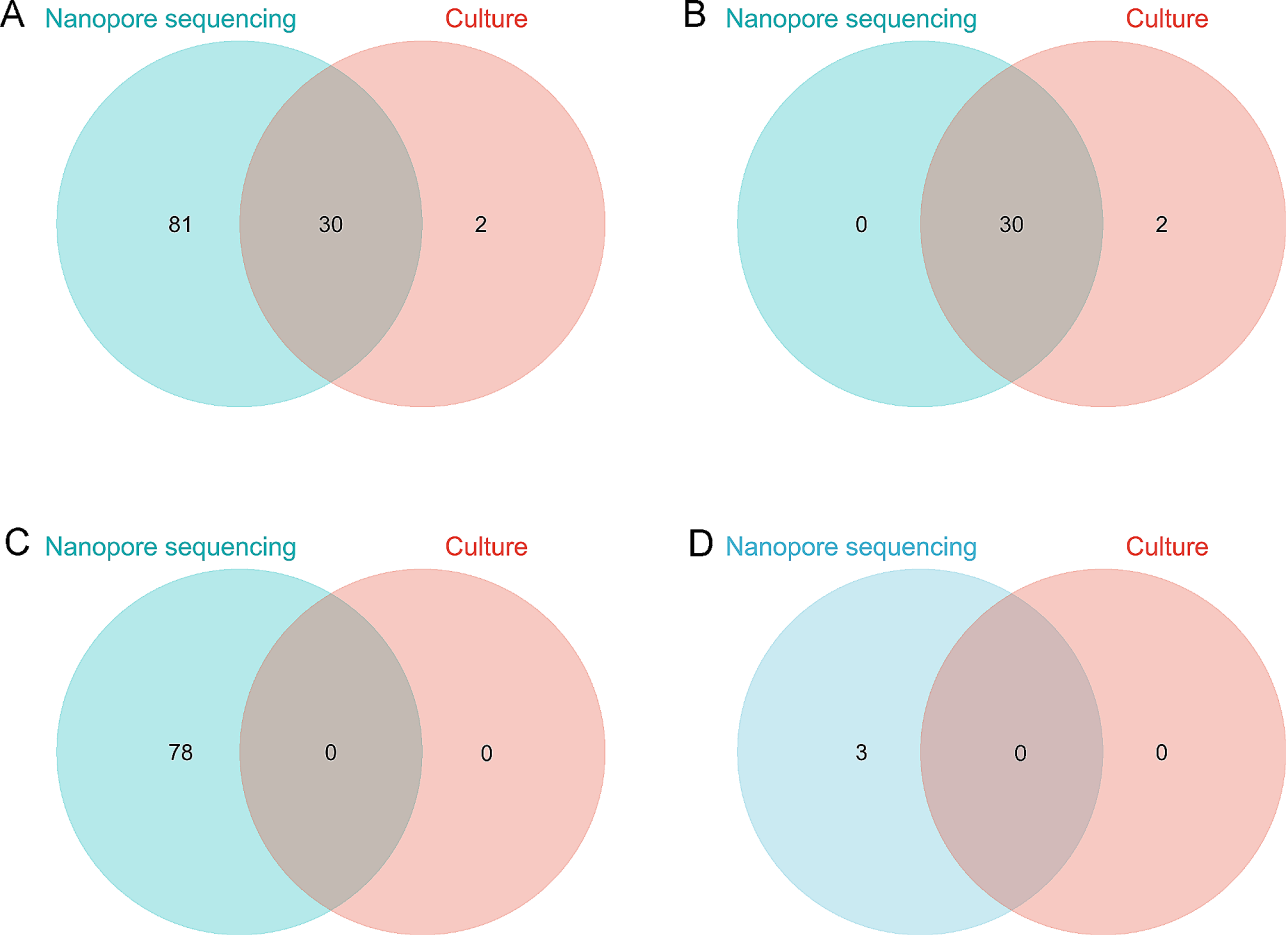
The distribution and overlap of positive results for the two tests in different diagnostic classifications. (**A**) all extrapulmonary tuberculosis, (**B**) confirmed extrapulmonary tuberculosis, (**C**) probable extrapulmonary tuberculosis, (**D**) non extrapulmonary tuberculosis

**Fig. 3:**
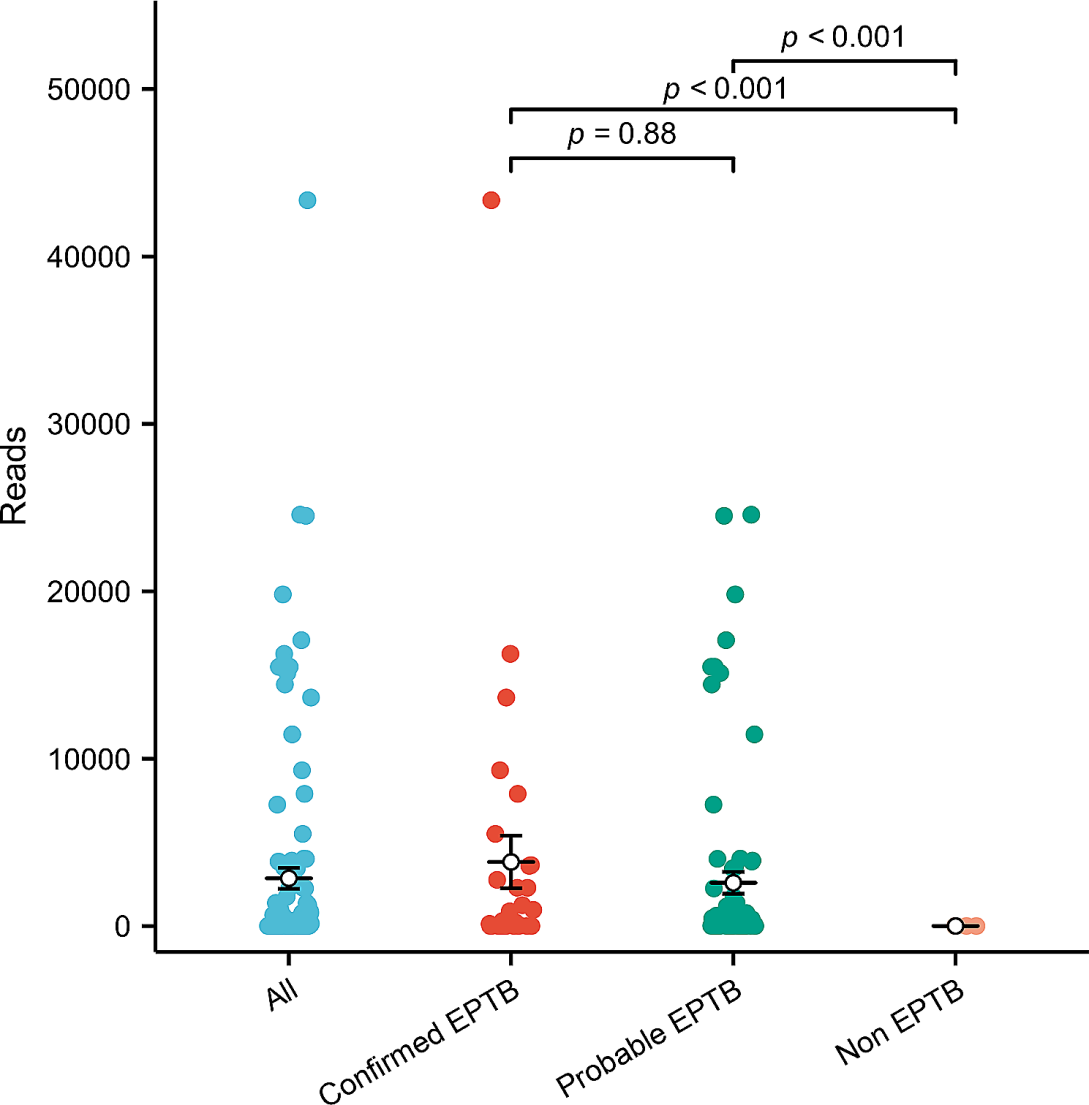
The distribution of reads for different classifications of positive nanopore sequencing patients

Based on the diagnostic classification, 32 cases of confirmed EPTB, 93 cases of probable EPTB, and 24 cases of non-EPTB were identified (Fig. 1). The number of positive results obtained using TNS in these three groups was 30, 78, and 3, respectively; the numbers obtained using AFB culture were 32, 0, 0, respectively (Fig. 1). The distribution and crossover of positive TNS and AFB culture with different diagnostic classifications are shown in Fig. 2. Among the whole sample, 111 were positive for EPTB using TNS and the distribution of TNS reads ranged from 1 to 43,360, with an average of 2851.0. The distribution of positive TNS reads in confirmed EPTB ranged from 2 to 43,360, with an average of 3830.0. The distribution of TNS positive reads in probable EPTB ranged from 1 to 24,572, with an average of 2583.9. The positive TNS reads in non-EPTB ranged from 2 to 4, with an average read of 3.0. A comparison of the positive TNS reads in confirmed EPTB with those in probable EPTB was not statistically significant (*P* > 0.05), whereas the positive reads in confirmed EPTB and probable EPTB were significantly higher than those in non-EPTB, and the difference was statistically significant (*P* < 0.001). The distribution and comparison of positive TNS reads for different diagnostic classifications are shown in Fig. 3. AFB smear was performed in only 82 of the patients in our sample and only two of these were positive.

### Diagnostic accuracy of TNS and AFB culture for EPTB

**Fig. 4:**
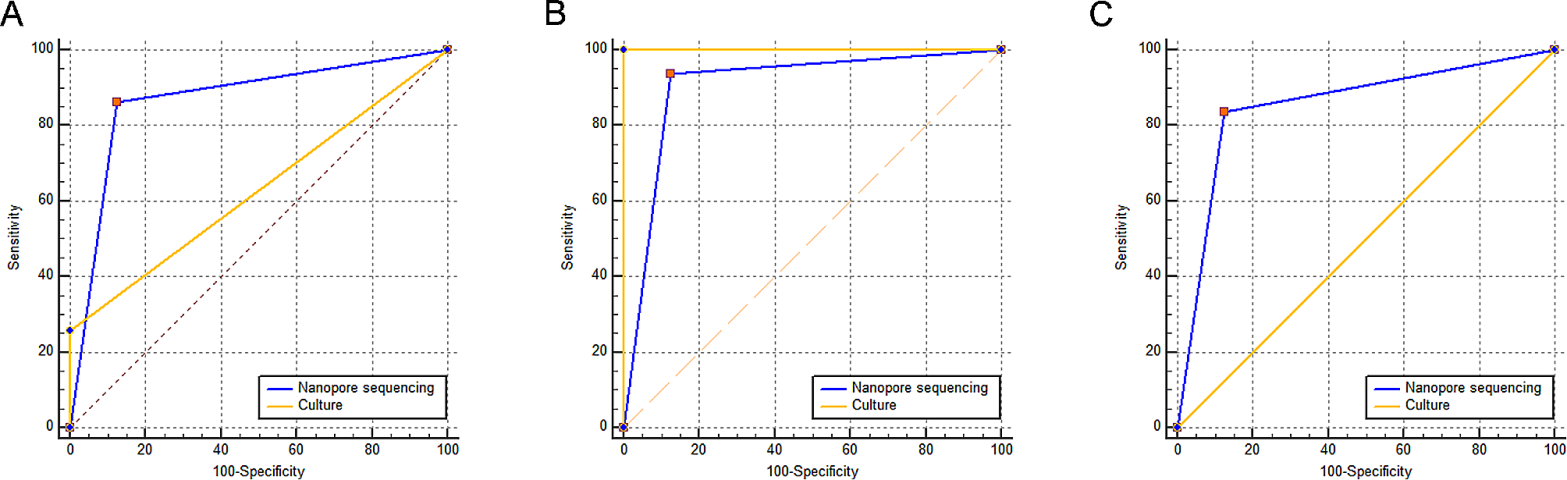
The receiver operating characteristic curves for the diagnosis of culture and nanopore sequencing for extrapulmonary tuberculosis in different diagnostic classifications. (**A**) all extrapulmonary tuberculosis, (**B**) confirmed extrapulmonary tuberculosis, (**C**) probable extrapulmonary tuberculosis

Using clinical diagnosis as the reference standard, the overall sensitivity, specificity, PPV, NPV, and AUC of TNS diagnosis of EPTB are displayed in Table 3. The receiver operating characteristic curves of TNS and AFB culture diagnoses of EPTB are shown in Fig. 4.

**Table 3:** Diagnostic accuracy of targeted nanopore sequencing and culture for extrapulmonary tuberculosis using clinical specimens

Patient group		EPTB	Non EPTB	Sensitivity (%)	Specificity (%)	PPV (%)	NPV (%)	AUC
Total EPTB								
TNS	+	108	3	86.4 (79.1–91.9)	87.5 (67.6–97.3)	97.3 (92.3–99.4)	55.3 (38.3–71.4)	0.87 (0.80–0.92)
	-	17	21					
Culture	+	32	0	25.6 (18.2–34.2)	100.0 (85.8–100.0)	100.0 (89.1–100.0)	20.5 (13.6–29.0)	0.63 (0.55–0.71)^a^
	-	93	24					
Confirmed EPTB								
TNS	+	30	3	93.8 (79.2–99.2)	87.5 (67.6–97.3)	90.9 (75.7–98.1)	91.3 (72.0-98.9)	0.91 (0.80–0.97)
	-	2	21					
Culture	+	32	0	100.0 (89.1–100.0)	100.0 (85.8–100.0)	100.0 (89.1–100.0)	100.0 (85.8–100.0)	1.00 (0.94-1.00)^b^
	-	0	24					
Probable EPTB								
TNS	+	78	3	83.9 (74.8–90.7)	87.5 (67.6–97.3)	96.3 (89.6–99.2)	58.3 (40.8–74.5)	0.86 (0.78–0.91)
	-	15	21					
Culture	+	0	0	0.0 (0.0-3.9)	100.0 (85.8–100.0)	ND	20.5 (13.6–29.0)	0.50 (0.41–0.59)^a^
	-	93	24					

Comparison of the diagnostic accuracy of nanopore sequencing and culture, ^a^*P* < 0.001, ^b^*P* = 0.209

EPTB, extrapulmonary tuberculosis; PPV, positive predictive value; NPV, negative predictive value; AUC, area under the curve; TNS, targeted nanopore sequencing; ND, not determined

The sensitivity, specificity, PPV, NPV, and AUC of TNS and AFB culture for confirmed EPTB and probable EPTB are also displayed in Table 3. The receiver operating characteristic curves of these two tests for confirmed EPTB and probable EPTB are shown in Fig. 4.

The diagnostic accuracy of TNS and AFB culture with different types of extrapulmonary specimens is shown in Table 4. Although some additional types of extrapulmonary specimens were used, the number of each of these was too small to be included in the analysis.

**Table 4:** Diagnostic accuracy of targeted nanopore sequencing and culture for different types of extrapulmonary tuberculosis using clinical specimens

Sample type	Test		EPTB	Non EPTB	Sensitivity (%)	Specificity (%)	PPV (%)	NPV (%)	AUC
Lymph node tissue	TNS	+	28	1	96.5 (82.2–99.9)	85.7 (42.1–99.6)	96.5 (82.2–99.9)	85.7 (42.1–99.6)	0.91 (0.77–0.98)^a^
		-	1	6					
	Culture	+	10	0	34.5 (17.9–54.3)	100.0 (59.0-100.0)	100.0 (69.2–100.0)	26.9 (11.6–47.8)	0.67 (0.50–0.82)^b^
		-	19	7					
Cerebrospinal fluid	TNS	+	16	1	66.7 (44.7–84.4)	75.0 (19.4–99.4)	94.1 (71.3–99.9)	27.3 (6.0–61.0)	0.71 (0.51–0.86)^c^
		-	8	3					
	Culture	+	9	0	37.5 (18.8–59.4)	100.0 (39.8–100.0)	100.0 (66.4–100.0)	21.1 (6.1–45.6)	0.69 (0.49–0.85)^d^
		-	15	4					
Pleural tissue	TNS	+	19	0	90.5 (69.6–98.8)	ND	100.0 (82.4–100.0)	0.0 (0.0-84.2)	ND
		-	2	0					
	Culture	+	1	0	4.8 (0.1–23.8)	ND	100.0 (2.5–100.0)	0.0 (0.0-16.8)	ND
		-	20	0					
Pleural effusion	TNS	+	17	0	85.0 (62.1–96.8)	100.0 (15.8–100.0)	100.0 (80.5–100.0)	40.0 (5.3–85.3)	0.93 (0.73–0.99)^e^
		-	3	2					
	Culture	+	5	0	25.0 (8.7–49.1)	100.0 (15.8–100.0)	100.0 (47.8–100.0)	11.8 (1.5–36.4)	0.63 (0.40–0.82)^b^
		-	15	2					

Comparison of the diagnostic accuracy of TNS and culture, ^b^*P* < 0.001, ^d^*P* = 0.902. Comparison of diagnostic accuracy of TNS in lymph node tissue and pleural effusion, ^a^*P* > 0.05. Comparison of diagnostic accuracy of TNS in cerebrospinal fluid and lymph node tissue and pleural effusion, ^c^*P* < 0.001. Comparison of diagnostic accuracy of AFB culture in lymph node tissue, cerebrospinal fluid and pleural effusion, ^e^*P* > 0.05

EPTB, extrapulmonary tuberculosis; PPV, positive predictive value; NPV, negative predictive value; AUC, area under the curve; TNS, targeted nanopore sequencing; ND, not determined

### Comparison of the diagnostic accuracy of TNS and AFB culture

Overall, the diagnostic accuracy of TNS was significantly better than that of AFB culture (*P* < 0.001) (Table 3). This was also true for probable EPTB alone. However, when cases of confirmed EPTB were analyzed separately, the accuracy of TNS was slightly lower than that of AFB culture, but the difference between the two was not statistically significant (*P* > 0.05) (Table 3). The diagnostic accuracy of TNS was superior to that of AFB culture for all of the extrapulmonary specimen types. The difference between the two methods was significant in lymph node tissue and pleural effusion (*P* < 0.001) (Table 4) but not in cerebrospinal fluid (*P* > 0.05) (Table 4). The diagnostic accuracy of TNS was similar for lymph node tissue and pleural effusion specimens (*P* > 0.05); while the diagnostic accuracy using cerebrospinal fluid specimens was moderate and significantly lower than for lymph node tissue and pleural effusion (*P* < 0.001) (Table 4). The accuracy of AFB culture using lymph node tissue, cerebrospinal fluid, and pleural effusion was similar, with no significant difference (*P* > 0.05) (Table 4).

## Discussion

Establishing a diagnosis of EPTB can be challenging [16]. One challenge is the difficulty in obtaining specimens for noninvasive testing [17]. Unlike PTB, which can be diagnosed using sputum samples, diagnosing EPTB requires specimens from affected areas of the body, such as the lymph nodes, bones, or central nervous system [18, 19]. These invasive procedures may be complex, require specialized equipment, and be uncomfortable for the patient. Another obstacle to EPTB diagnosis is the paucibacillary nature of the specimens obtained [20]. The number of TB bacteria present in these specimens is usually very low, requiring more sensitive diagnostic methods [20]. The development of more sensitive diagnostic methods would also reduce the number of invasive procedures and improve patient satisfaction. The low bacterial load in specimens reduced the accuracy of conventional diagnostic methods, leading to potential misdiagnosis or delayed diagnosis [21]. In the present study, AFB smear of only 2.4% (2/82) of the specimens produced positive results. The low sensitivity of AFB smear means that they are rarely done as a preferred test when the specimen volume is limited. It was for this reason that AFB smear was not performed on all specimens in this study and why the other tests were not compared with AFB smear. AFB culture, on the other hand, is a crucial test in the diagnosis of TB. The AFB culture from 21.5% (32/149) of the specimens in this study were positive, supporting the characterization of extrapulmonary specimens as less bacterial. AFB culture is the gold standard for the diagnosis of TB, but the paucibacillary nature of EPTB makes AFB culture defective in the diagnosis of EPTB, and may miss part of the diagnosis of EPTB, in other words, a negative AFB culture does not completely exclude the diagnosis of EPTB [22]. Clinical diagnosis requires consideration of multiple factors to ensure all true positive cases are identified and to maximize specificity. However, clinical diagnosis itself also has the potential to reduce specificity and increase false-negative results. The positivity rate of the AFB culture in this study was low and could not effectively differentiate between EPTB [22]. Therefore, we chose clinical diagnosis as our diagnostic benchmark. Improvements in diagnostic technologies and techniques continue to be developed to address these challenges and provide more accurate and rapid diagnoses of EPTB, allowing for the timely initiation of appropriate treatment and improved patient outcomes.

Nanopore sequencing is a new generation of sequencing technology that has shown advantages in the diagnosis of various types of tumors and infectious diseases [23, 24]. It has also shown satisfactory results in the rapid diagnosis of TB [13]. Nanopore sequencing showed a significant improvement in accuracy in the diagnosis of TB compared to conventional AFB culture (AUC: 0.79 vs. 0.96; *P* < 0.05) [13]. TNS was developed to improve diagnostic detection in diseases with low bacterial content through the targeting of pathogens via PCR amplification [25]. TNS performed PCR amplification of the target pathogens prior to nanopore sequencing, which increased the DNA or RNA content of the target pathogens and thus may further improve the sensitivity of nanopore sequencing. RpoB is a conserved sequence in *Mycobacterium* spp. and can be efficiently used to amplify *Mycobacterium* spp (including TB and non-tuberculosis mycobacteria) and for strain identification [26]. Our results show that, overall, the accuracy of TNS for diagnosing EPTB is good and significantly higher than that of AFB culture. We found 17 false negative specimens (2 cases in the confirmed EPTB group and 15 cases in the probable EPTB group), and the specimen types were mainly cerebrospinal fluid and plasma membrane cavity fluid. As the levels of TB bacteria in these specimens were lower than those found in tissue and pus specimens, TNS still failed to detect TB. Three false positive specimens (all in non-EPTB group) were found, which may have been due to abnormalities in the PCR process, leading to contamination of the samples. When interpreting a TNS report, it is important to focus not only on the positive-negative results, but also on the sequence reads that correspond to the positive results. Our findings suggested that the reads in the confirmed and probable EPTB groups were significantly higher than those in the non-EPTB group, and the higher the reads the higher the likelihood of true positive. The TNS reads of these three false positive cases were very low, significantly lower than the reads of true positive specimens, suggesting a need to combine other factors to evaluate TNS results with low reads.

For the confirmed EPTB group, AFB culture was the reference standard. In this group, the diagnostic accuracy of TNS was excellent. In the probable EPTB group, AFB culture was ineffective for the diagnosis of EPTB, but TNS still demonstrated very good diagnostic accuracy with this group of patients. The value of TNS for use with this latter type of patient is much greater.

EPTB can infect any organ outside the lungs, and the symptoms that characterize infections at different sites can vary, as can the specimens required from them [27]. For example, the primary specimen for tuberculous meningitis is cerebrospinal fluid; for lymph node TB, the specimen may be pus or tissue; and for tuberculous pleurisy, the specimen may be pleural fluid or pleural tissue. The most common EPTBs include tuberculous pleurisy, lymph node TB, tuberculous meningitis, and bone and joint TB. Eighteen types of extrapulmonary specimens were included in this study, the most common being lymph node tissue, cerebrospinal fluid, pleural effusion, and pleural tissue. A subgroup analysis found the diagnostic accuracy of TNS using lymph node tissue and pleural effusion specimens to be excellent. Our results were superior to those obtained using other molecular diagnostic tests, especially with pleural effusion specimens. Pleural effusions are considered to be extremely low in TB bacteria, and other molecular tests such as Xpert MTB/RIF are poor detectors when using pleural effusions [28]. It is hoped that our results will provide an efficient and accurate option for the diagnosis of TB pleurisy. We found the detection ability of TNS using cerebrospinal fluid to be moderate. However, its ability to detect TB bacteria in cerebrospinal fluid was still superior to that of Xpert MTB/RIF. TNS may be a potentially efficient detection tool for TB meningitis. However, the sample sizes of the various subgroups of the current study were limited; therefore, these results should be treated with caution. Further studies with larger sample sizes are needed to verify the diagnostic accuracy of TNS in different types of extrapulmonary specimens.

Our research had several limitations. First, this was a single-center retrospective study; therefore, the patient selection process may have introduced bias. Consequently, the results may not fully represent the actual population affected by the disease. Second, the study was conducted in an area with a high burden of TB. This may have affected the generalizability of the findings. Third, we focused solely on assessing the accuracy of TNS diagnosis of EPTB. The presence of drug resistance was not investigated. Lastly, our sample size was limited, and this, in turn, limited the number of different types of extrapulmonary specimens analyzed. In view of the limited sample size, confirmation of our results by larger studies with more diverse EPTB patient populations is warranted.

## Conclusions

TNS demonstrates good diagnostic accuracy for EPTB and rapid results. It also showed very good diagnostic accuracy across different types of extrapulmonary specimens. TNS may become a new option for the efficient diagnosis of EPTB.

## Data Availability

Data will be available from the corresponding author on reasonable request.

## References

[1] World Health Organization. Global Tuberculosis Report 2023. 2023.

[2] Lee JY. Diagnosis and treatment of extrapulmonary tuberculosis. Tuberc Respir Dis (Seoul). 2015;78(2):47–55. 10.4046/trd.2015.78.2.47 25861336 10.4046/trd.2015.78.2.47 PMC4388900

[3] Yu G, Shen Y, Ye B, Chen D, Xu K. Comparison of CapitalBio™ Mycobacterium nucleic acid detection test and Xpert MTB/RIF assay for rapid diagnosis of extrapulmonary tuberculosis. J Microbiol Methods. 2020;168:105780. 10.1016/j.mimet.2019.105780 31751598 10.1016/j.mimet.2019.105780

[4] Mohammed H, Assefa N, Mengistie B. Prevalence of extrapulmonary tuberculosis among people living with HIV/AIDS in sub-saharan Africa: a systemic review and meta-analysis. HIV AIDS (Auckl). 2018;10:225–37. 10.2147/hiv.S176587 30464643 10.2147/hiv.S176587 PMC6225852

[5] Baykan AH, Sayiner HS, Aydin E, Koc M, Inan I, Erturk SM. Extrapulmonary tuberculosıs: an old but resurgent problem. Insights Imaging. 2022;13(1):39. 10.1186/s13244-022-01172-0 35254534 10.1186/s13244-022-01172-0 PMC8901940

[6] Yu G, Zhong F, Zhao W, Ye B, Xu K, Chen G. Head-to-head comparison of the diagnostic value of five tests for constrictive tuberculous pericarditis: five tests for constrictive TBP. Int J Infect Dis. 2022;120:25–32. 10.1016/j.ijid.2022.04.018 35429643 10.1016/j.ijid.2022.04.018

[7] Slail MJ, Booq RY, Al-Ahmad IH, Alharbi AA, Alharbi SF, Alotaibi MZ, et al. Evaluation of Xpert MTB/RIF Ultra for the diagnosis of Extrapulmonary Tuberculosis: a retrospective analysis in Saudi Arabia. J Epidemiol Glob Health. 2023. 10.1007/s44197-023-00150-z 37707714 10.1007/s44197-023-00150-z PMC10686912

[8] Chattopadhyay S, Biswas T, Banerjee A, Chaudhury N, Mondal R, Nath A. Diagnostic Approach to Extrapulmonary Tuberculosis by Cartridge-based nucleic acid amplification test. J Assoc Physicians India. 2023;71(6):11–2. 10.5005/japi-11001-0260 37355841 10.5005/japi-11001-0260

[9] Dahiya B, Mehta N, Soni A, Mehta PK. Diagnosis of extrapulmonary tuberculosis by GeneXpert MTB/RIF Ultra assay. Expert Rev Mol Diagn. 2023;23(7):561–82. 10.1080/14737159.2023.2223980 37318829 10.1080/14737159.2023.2223980

[10] Shen Y, Fang L, Ye B, Xu X, Yu G, Zhou L. The role of Core Needle Biopsy Pathology combined with molecular tests in the diagnosis of Lymph Node Tuberculosis. Infect Drug Resist. 2022;15:335–45. 10.2147/idr.S350570 35140479 10.2147/idr.S350570 PMC8818765

[11] Kolmogorov M, Billingsley KJ, Mastoras M, Meredith M, Monlong J, Lorig-Roach R, et al. Scalable nanopore sequencing of human genomes provides a comprehensive view of haplotype-resolved variation and methylation. Nat Methods. 2023. 10.1038/s41592-023-01993-x 37710018 10.1038/s41592-023-01993-x PMC11222905

[12] Chen J, Xu F. Application of Nanopore Sequencing in the diagnosis and treatment of pulmonary infections. Mol Diagn Ther. 2023. 10.1007/s40291-023-00669-8 37563539 10.1007/s40291-023-00669-8 PMC10590290

[13] Yu G, Shen Y, Zhong F, Zhou L, Chen G, Fang L, et al. Diagnostic accuracy of nanopore sequencing using respiratory specimens in the diagnosis of pulmonary tuberculosis. Int J Infect Dis. 2022;122:237–43. 10.1016/j.ijid.2022.06.001 35671950 10.1016/j.ijid.2022.06.001

[14] Deng Q, Cao Y, Wan X, Wang B, Sun A, Wang H, et al. Nanopore-based metagenomic sequencing for the rapid and precise detection of pathogens among immunocompromised cancer patients with suspected infections. Front Cell Infect Microbiol. 2022;12:943859. 10.3389/fcimb.2022.943859 36204638 10.3389/fcimb.2022.943859 PMC9530710

[15] Yu G, Lin T, Yu Y, Chen P, Chen M, Zhang Y, et al. Application of Mycobacterium tuberculosis RNA for the Rapid diagnosis of Lymph Node Tuberculosis using different specimens. Infect Drug Resist. 2023;16:179–87. 10.2147/idr.S392045 36636372 10.2147/idr.S392045 PMC9831075

[16] Rao PD, Devi DRG, Gouri SRM, Arjun AS, Krishnappa L, Azeem A. Evaluation of immunohistochemistry technique for diagnosis of Extrapulmonary Tuberculosis in Biopsy tissue specimen as compared to Composite Diagnostic Criteria. J Glob Infect Dis. 2022;14(4):136–41. 10.4103/jgid.jgid_112_22 36636303 10.4103/jgid.jgid_112_22 PMC9831204

[17] Yu G, Wang L, Shen Y, Fang L, Yang J, Ye B, et al. Comparison of the Diagnostic Accuracy of Xpert MTB/RIF and CapitalBio Mycobacterium RT-PCR detection assay for tuberculous pericarditis. Infect Drug Resist. 2022;15:2127–35. 10.2147/idr.S360064 35498628 10.2147/idr.S360064 PMC9041359

[18] Yu G, Wang X, Zhu P, Shen Y, Zhao W, Zhou L. Comparison of the efficacy of metagenomic next-generation sequencing and Xpert MTB/RIF in the diagnosis of tuberculous meningitis. J Microbiol Methods. 2021;180:106124. 10.1016/j.mimet.2020.106124 33321144 10.1016/j.mimet.2020.106124

[19] Shen Y, Yu G, Zhong F, Kong X. Diagnostic accuracy of the Xpert MTB/RIF assay for bone and joint tuberculosis: a meta-analysis. PLoS ONE. 2019;14(8):e0221427. 10.1371/journal.pone.0221427 31437232 10.1371/journal.pone.0221427 PMC6705841

[20] Uddin MKM, Ather MF, Kabir S, Rahman A, Choudhury S, Nasrin R, et al. Diagnostic performance of different laboratory methods for the detection of Extrapulmonary Tuberculosis. Microorganisms. 2023;11(4). 10.3390/microorganisms11041066 10.3390/microorganisms11041066 PMC10142428 37110489

[21] Kang W, Yu J, Liang C, Wang Q, Li L, Du J, et al. Epidemiology and Association Rules Analysis for pulmonary tuberculosis cases with Extrapulmonary tuberculosis from age and gender perspective: a large-scale Retrospective Multicenter Observational Study in China. Int J Clin Pract. 2023;2023:5562495. 10.1155/2023/5562495 37609664 10.1155/2023/5562495 PMC10442182

[22] Yu G, Ye B, Chen D, Zhong F, Chen G, Yang J, et al. Comparison between the diagnostic validities of Xpert MTB/RIF and interferon-γ release assays for tuberculous pericarditis using pericardial tissue. PLoS ONE. 2017;12(12):e0188704. 10.1371/journal.pone.0188704 29211755 10.1371/journal.pone.0188704 PMC5718425

[23] Zheng P, Zhou C, Ding Y, Liu B, Lu L, Zhu F et al. Nanopore sequencing technology and its applications. MedComm (2020). 2023;4(4):e316. 10.1002/mco2.316 10.1002/mco2.316 PMC10333861 37441463

[24] Papatsirou M, Scorilas A, Sideris DC, Kontos CK. Targeted nanopore sequencing for the identification of novel PRMT1 circRNAs unveils a diverse transcriptional profile of this gene in breast cancer cells. Genes Dis. 2024;11(2):589–92. 10.1016/j.gendis.2023.04.013 37692475 10.1016/j.gendis.2023.04.013 PMC10491911

[25] Wang M, Fu A, Hu B, Tong Y, Liu R, Liu Z, et al. Nanopore targeted sequencing for the Accurate and Comprehensive Detection of SARS-CoV-2 and other respiratory viruses. Small. 2020;16(32):e2002169. 10.1002/smll.202002169 32578378 10.1002/smll.202002169 PMC7361204

[26] Hashemzadeh M, Dezfuli AA, Khosravi AD, Bandbal MM, Ghorbani A, Hamed M, et al. Molecular identification of non-tuberculous mycobacterial species isolated from extrapulmonary samples using real-time PCR and rpoB sequence analysis. AMB Express. 2023;13(1):43. 10.1186/s13568-023-01553-8 37147556 10.1186/s13568-023-01553-8 PMC10163175

[27] Yu G, Zhong F, Ye B, Xu X, Chen D, Shen Y. Diagnostic accuracy of the Xpert MTB/RIF Assay for Lymph Node Tuberculosis: a systematic review and Meta-analysis. Biomed Res Int. 2019;2019:4878240. 10.1155/2019/4878240 31236407 10.1155/2019/4878240 PMC6545759

[28] Qiu YR, Chen YY, Wu XR, Li YP, Cao XJ, Yu ZY, et al. Accuracy of Xpert MTB/RIF assay for the diagnosis of tuberculous pleural effusion. J Clin Lab Anal. 2022;36(1):e24185. 10.1002/jcla.24185 34919739 10.1002/jcla.24185 PMC8761404

